# Transcriptome profiling reveals potential genes involved in browning of fresh-cut eggplant (*Solanum melongena* L.)

**DOI:** 10.1038/s41598-021-94831-z

**Published:** 2021-08-09

**Authors:** Xiaohui Liu, Aidong Zhang, Jie Zhao, Jing Shang, Zongwen Zhu, Xuexia Wu, Dingshi Zha

**Affiliations:** 1 Horticultural Research Institute, Shanghai Academy of Agricultural Sciences, Shanghai, 201403 China; 2 Shanghai Key Laboratory of Protected Horticultural Technology, Shanghai, 201403 China; 3 Pudong New District Agro-Technology Extension Center, Shanghai, 201201 China; 4 College of Food Science, Shanghai Ocean University, Shanghai, 201306 China

**Keywords:** Plant breeding, Plant sciences, Plant molecular biology

## Abstract

Fresh-cut processing promotes enzymatic browning of fresh fruits and vegetables, which negatively affects the product appearance and impacts their nutrition. We used RNA-sequencing to analyze the transcriptomic changes occurring during the browning of fresh-cut eggplant fruit samples from both browning-sensitive and browning-resistant cultivars to investigate the molecular mechanisms involved in browning. A total of 8347 differentially expressed genes were identified, of which 62 genes were from six gene families (i.e., *PPO*, *PAL*, *POD*, *CAT*, *APX*, and *GST*) potentially associated with enzymatic browning. Furthermore, using qRT-PCR, we verified 231 differentially regulated transcription factors in fresh-cut eggplant fruits. The enzyme activities of PPO, POD, PAL, and CAT in ‘36’ were significantly higher than those of ‘F’ fresh-cut for 15 min. Both PPO and POD play a major role in the browning of eggplant pulp and might therefore act synergistically in the browning process. Meanwhile, qPCR results of 18 browning related genes randomly screened in 15 eggplant materials with different browning tolerance showed variant-specific expression of genes. Lastly, gene regulatory networks were constructed to identify the browning-related genes. This work provides a basis for future molecular studies of eggplants, and lays a theoretical foundation for the development of browning-resistant fresh-cut fruits and vegetables.

## Introduction

*Solanum melongena* L., commonly known as eggplant, is an essential vegetable crop. It is a good source of dietary minerals, vitamins, and anthocyanins, with a high oxygen radical absorbance capacity and low caloric value^1^. Eggplant has been shown to have important cardiovascular protecting, anticancer, and anti-aging effects^2^. However, fresh-cut eggplants brown easily which can negatively affect their flavor, odor, nutrients, and commercial value. Browning has become one of the most vital limitations in eggplant processing. Therefore, understanding the mechanism of enzymatic browning and controlling the occurrence of browning have become the focus of research in the fresh-cut fruit and vegetable industry. Furthermore, breeding new eggplant varieties resistant to browning has become an important method to fundamentally solve the browning of eggplant fruit.

Browning, a common post-harvest problem for many fruits and vegetables, can be divided into two categories: enzymatic and non-enzymatic browning^3^. Non-enzymatic browning occurs as a result of various chemical reactions such as Maillard reactions, caramelization, oxidation of vitamin C, and polyphenol polymerization in daily life^4^. Enzymatic browning is considered as the main reaction that causes browning in post-harvest storage and processing of fruits and vegetables, and has been the focus of postharvest research^5^. It refers to the physiological and biochemical processes in which phenolic substances in plant organs or tissues are oxidized to form quinones under the action of polyphenol oxidase under aerobic conditions^6^. Quinones then polymerize to form brown or black substances, resulting in tissue discoloration. Enzymatic browning occurs only in the presence of phenols, enzymes, and oxygen^7^. As an important substrate for enzymatic browning of fruit and vegetable tissues, phenols are widely distributed in the roots, stems, leaves, flowers, and fruits of plants—with varying abundance between different types of plants^8^. Phenolic compounds are important secondary metabolites in plants, which are synthesized mainly through phenylalanine metabolic pathways^9^. Phenylalanine ammonia-lyase (PAL) is the starting enzyme of the phenylpropane metabolic pathway^10^. The phenylalanine amino group is deaminated by PAL to form trans-cinnamic acid, which is hydroxylated by cinnamate-4-hydroxylase (C4H) to form trans-4-coumaric acid. Then, under the actions of coumarate 3-hydroxylase (C3H), 4-coumarate-CoA ligase (4CL), hydroxycinnamoyl-CoA: shikimate/quinate hydroxy cinnamoyl transferase (HCT), chalcone synthase (CHS), cinnamoyl-CoA reductase (CCR), and other enzymes, it enters the metabolic pathways of lignin synthesis, chlorogenic acid synthesis, and flavonoid (anthocyanin, procyanidin, rutin) synthesis^11^. Furthermore, enzymatic browning is mostly a result of transforming phenols into o-quinones—catalyzed by polyphenol oxidase (PPO)^12^. Other enzymes, such as peroxidase (POD), catalase (CAT), superoxide dismutase (SOD), ascorbate peroxidase (APX), and glutathione S-transferase (GST) also play a role in inducing or inhibiting the enzymatic browning of fresh-cut fruits and vegetables^13^. Therefore, it is essential to study these genes that encode key enzymes in eggplant enzymatic browning and phenolic compound formation, which may lay the foundation for better understanding of the browning mechanism in fresh-cut fruits and vegetables.

With the development of high-throughput sequencing, technologies such as genomics, transcriptomics, proteomics, and metabolomics have been widely used to study specific biological processes and molecular mechanisms of organisms^14^. In recent years, RNA-sequencing (RNA-seq) as an efficient and fast transcriptome research method has changed people's understanding of transcriptomics, and has been increasingly used to explore differences in gene expression in plants, such as licorice (*Glycyrrhiza glabra* L.)^15^, luffa (*Luffa cylindrica* (L.) Roem.) ^16^, potato (*Solanum tuberosum* L.) ^17^, strawberry (*Fragaria ananassa *Duch)^18^, and pear (*Pyrus betulifolia* Bunge)^19^. In a study by Zhu et al*.* on fresh-cut luffa browning, they found that there were 15 genes with significantly different expressions in enzymatic browning, including *PPO*, *POD*, *PAL*, *CAT*, and 4 *WRKY* transcription factors (TFs)^20^. However, there are few reports on the browning mechanism of fresh-cut eggplant fruit.

We identified two cultivars of eggplant materials, ‘F’ and ‘36’, as either resistant or sensitive to browning, respectively, by fresh-cutting more than 60 eggplant materials and recording their respective browning times. In the current study, we aimed to (1) conduct transcriptomic profiling of the two types of materials, (2) identify the key differentially expressed genes (DEGs) related to enzymatic browning and phenolic compound formation, and (3) provide new insights into the molecular mechanisms of eggplant browning.

## Results

### Overview of transcriptomic data for cultivars ‘F’ and ‘36’

**Figure 1:**
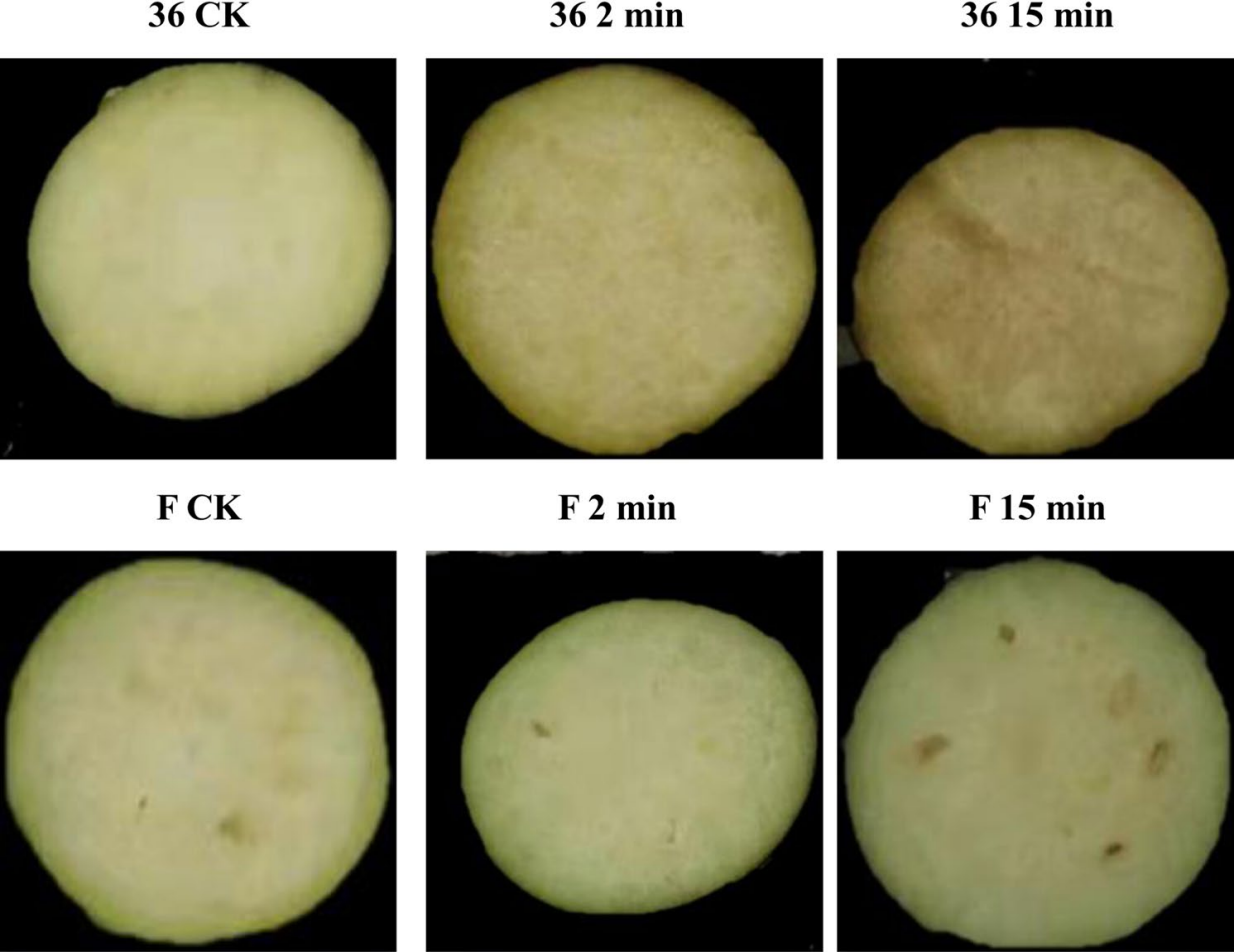
The browning degree of browning-sensitive (36) and browning-resistant (F) cultivars.

**Figure 2:**
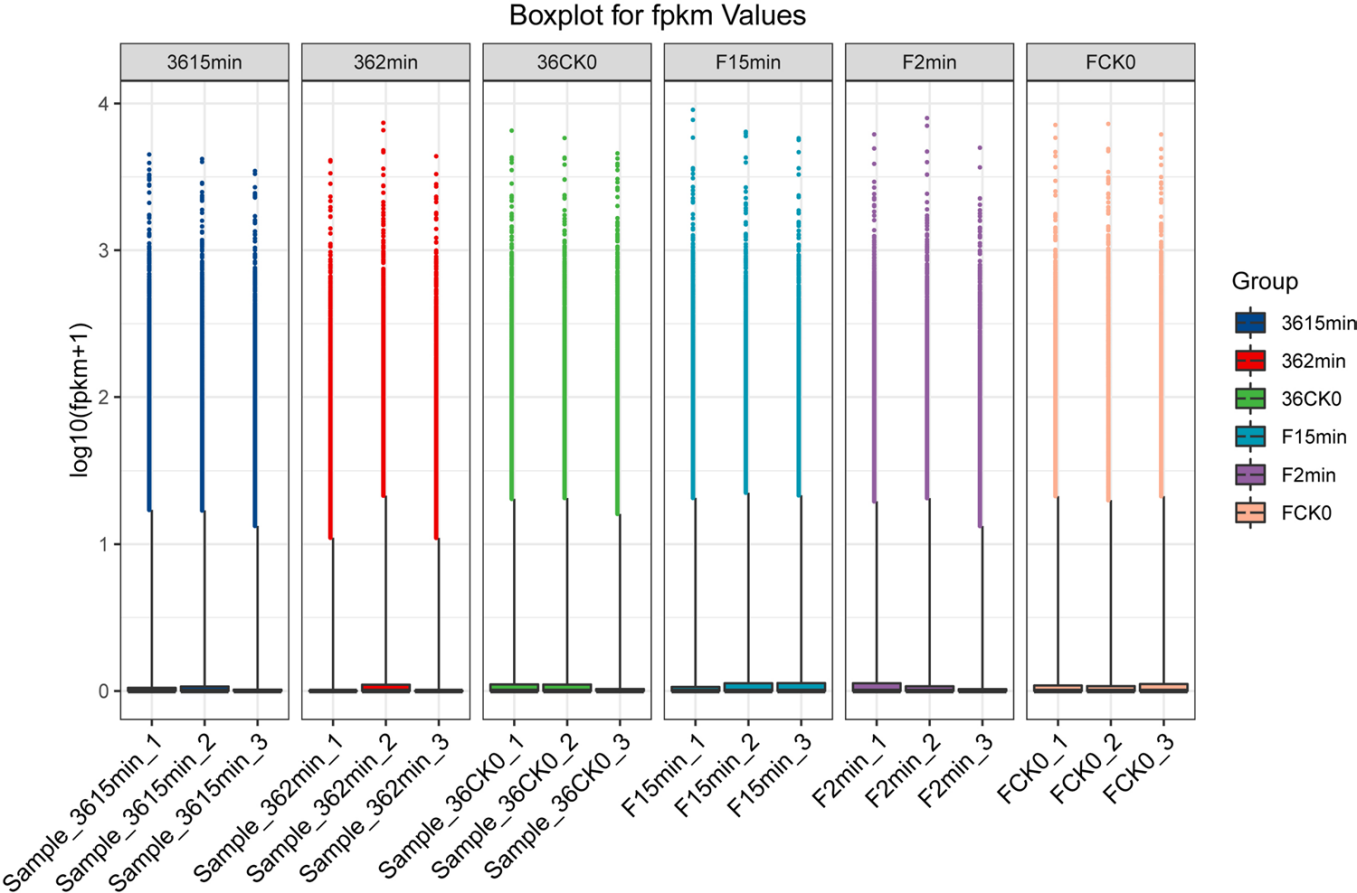
Distribution of gene expression levels in each sample. FPKM, fragments per kb per million.

The browning-sensitive cultivar (‘36’) and browning-resistant cultivar (‘F’) of eggplant showed different degrees of browning with treatment time (Fig. 1). As shown in Table 1, a total of 111.55 Gb clean data were obtained after 18 samples were analyzed and sequenced using a reference transcriptome (ftp://ftp.kazusa.or.jp/pub/eggplant/SME_r2.5.1.fa.gz). The effective data volume of each sample was 5.73–7.04 Gb; 93.61–94.50% of bases had a quality above Q30, and the average GC content was 45.56%. Reads were compared to the reference genome to obtain the genome alignment of each sample, with an alignment rate of 83.50–95.14%. We also analyzed the distribution of gene expression levels in each sample (Fig. 2). We determined the minimum, the first quartile (25%), the median (50%), the third quartile (75%), and the maximum value of gene expression level for each sample. The symmetry and degree of dispersion of the data distribution were observed, with remarkably high gene expression levels. Heatmap of Pearson's correlation coefficients between all pairs of samples is shown in Supplementary Figure S1.

**Table 1:** Statistics of clean reads in the eggplant transcriptomes.

Sample	raw_reads	raw_bases	clean_reads	clean_bases	valid_bases (%)	Q30 (%)	GC (%)
F 15min_1	49.75M	7.46G	48.83M	6.63G	88.89	94.15	44.08
F 15min_2	43.77M	6.57G	42.98M	5.84G	88.89	93.84	43.55
F 15min_3	43.15M	6.47G	42.38M	5.77G	89.21	94.08	43.46
F 2min_1	50.03M	7.50G	49.08M	6.54G	87.16	93.68	43.82
F 2min_2	48.79M	7.32G	47.97M	6.49G	88.66	94.36	43.43
F 2min_3	49.04M	7.36G	48.16M	6.52G	88.62	94.20	48.59
F CK0_1	45.73M	6.86G	44.96M	6.11G	89.06	94.20	43.50
F CK0_2	47.44M	7.12G	46.49M	6.20G	87.17	93.87	44.36
F CK0_3	44.35M	6.65G	43.44M	5.75G	86.47	93.61	44.04
36 15min_1	43.53M	6.53G	42.75M	5.81G	89.00	94.33	46.06
36 15min_2	44.28M	6.64G	43.46M	5.95G	89.60	94.04	46.80
36 15min_3	44.47M	6.67G	43.65M	5.89G	88.30	94.31	49.09
36 2min_1	49.59M	7.44G	48.75M	6.57G	88.30	94.50	50.06
36 2min_2	43.62M	6.54G	42.83M	5.73G	87.57	94.07	43.78
36 2min_3	46.19M	6.93G	45.34M	6.05G	87.39	94.01	49.84
36 CK0_1	48.12M	7.22G	47.28M	6.33G	87.74	94.03	44.55
36 CK0_2	52.72M	7.91G	51.84M	7.04G	89.07	94.40	44.25
36 CK0_3	47.98M	7.20G	47.12M	6.33G	88.01	94.02	46.79

### Identification and classification of DEGs

**Figure 3:**
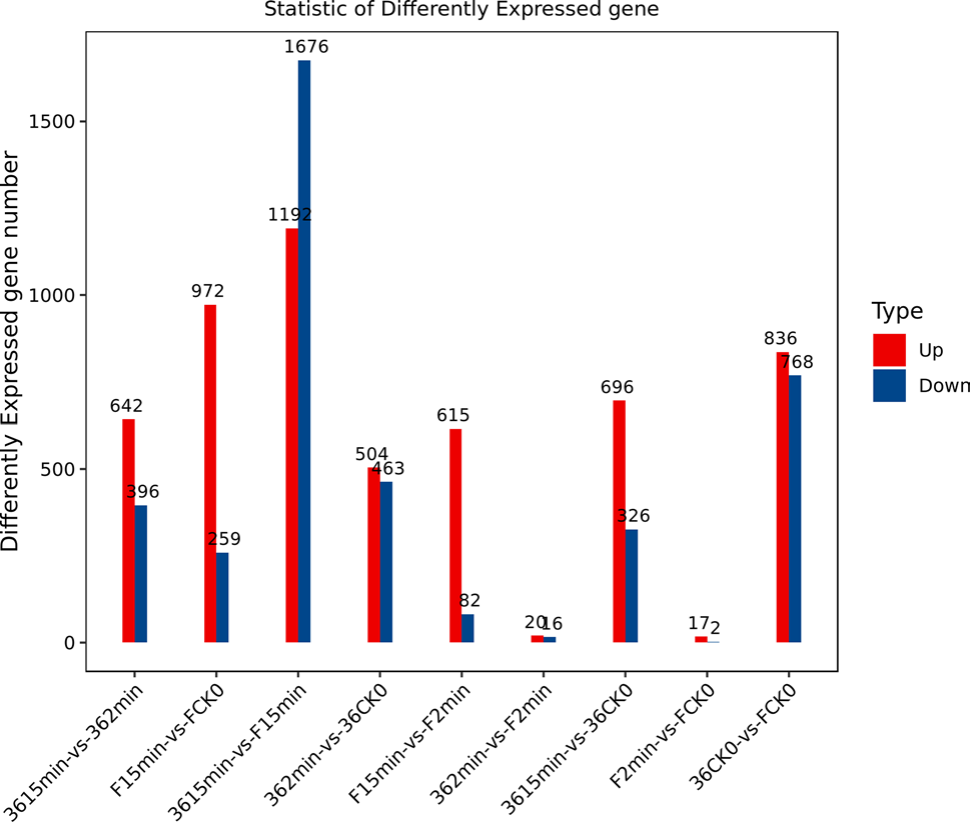
Differentially expressed genes (DEGs) in each comparison group.

Identification of DEGs between the two eggplant cultivars was one of the aims of the present study. To achieve this goal, we compared the transcriptional profiles of cultivars ‘36’ and ‘F’, which were respectively used as a test and a control for screening DEGs. According to the standard criteria (fold change > 2; *p*-value < 0.05), we analyzed the difference in the expression of the same gene between treatment pairs. For cultivar ‘36’, we identified 4095 DEGs between fresh-cut for 2 min and CK0 (control before fresh-cut) (1971 up- and 2124 down-regulated genes), 2212 DEGs between 15 min and CK0 (857 up- and 1355 down-regulated genes), and 1957 DEGs between 15 and 2 min (996 up- and 961 down-regulated genes). For cultivar ‘F’, we identified 20 DEGs between fresh-cut for 2 min and CK0 (11 up- and 9 down-regulated genes), 1313 DEGs between 15 min and CK0 (1188 up- and 125 down-regulated genes), and 1087 DEGs between 15 and 2 min (1014 up- and 73 down-regulated genes). Furthermore, there were 2299 DEGs between the CK0 of ‘36’ and ‘F’ (1363 up- and 936 down-regulated genes), 2810 DEGs between fresh-cut for 2 min of ‘36’ and ‘F’ (1633 up- and 1177 down-regulated genes), and 3820 DEGs between fresh-cut for 15 min of ‘36’ and ‘F’ (1336 up- and 2484 down-regulated genes) (Fig. 3, Supplementary Table S1). The DEGs were then subjected to Gene Ontology (GO) and Kyoto Encyclopedia of Genes and Genomes (KEGG) pathway enrichment analyses (Supplementary Table S2–S3).

### Functional annotation and classification of DEGs

**Figure 4:**
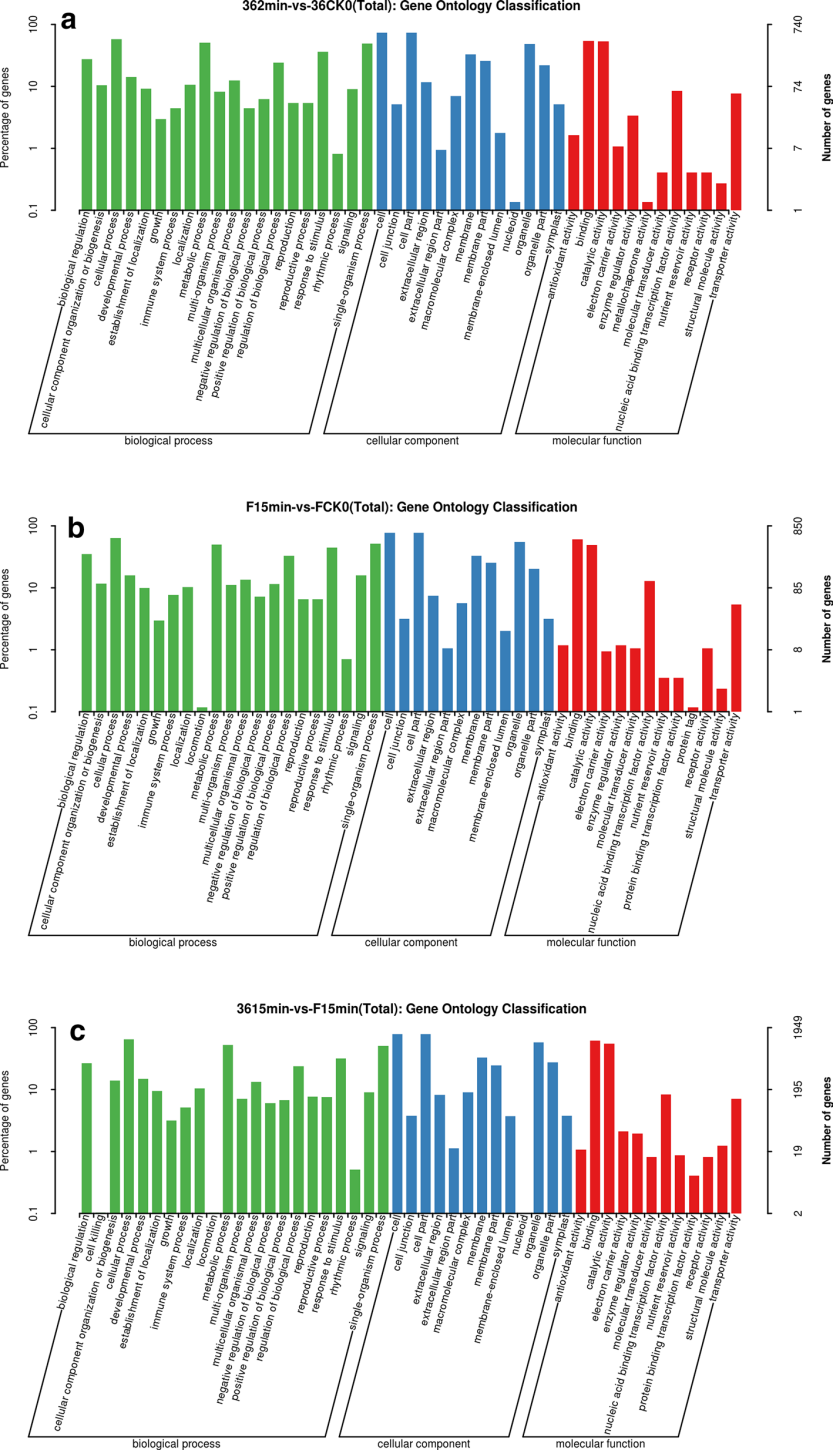
(**a**) GO functional enrichment of the ‘36’ 2 min vs ‘36’ CK0 groups. (**b**) GO functional enrichment of the ‘F’ 15 min vs ‘F’ CK0 groups. (**c**) GO functional enrichment of the ‘36’ 15 min vs ‘F’ 15 min groups. The figures was drawn with DEseq (v3.5.1). (http://www.bioconductor.org/packages/3.8/bioc/html/DESeq.html).

To determine the function of DEGs, GO and KEGG pathway analyses were carried out. For cultivar ‘36’, the treatment pair with the most DEGs was the 2 min/CK0 pair. In this treatment pair, GO functional enrichment analysis was carried out (Fig. 4a). “Metabolic process,” “cell,” and “binding” were the dominant terms in the “biological process,” “cellular component,” and “molecular function” categories, respectively. We also identified a relatively large number of genes associated with “catalytic activity,” “antioxidant activity,” “biological regulation,” “response to stimulus,” “single-organism process,” and “cellular process,” with only a few genes related to “cell killing,” “nucleoid,” and “protein binding TF activity.” As shown in Fig. 4b,c, GO functional enrichment analysis of the treatment pairs ‘F’_15 min_/‘F’_CK0_ and ‘36’_15 min_/‘F’_15 min_ showed similar results as the ‘36’_2 min_/‘36’_CK0_ treatment pair.

**Figure 5:**
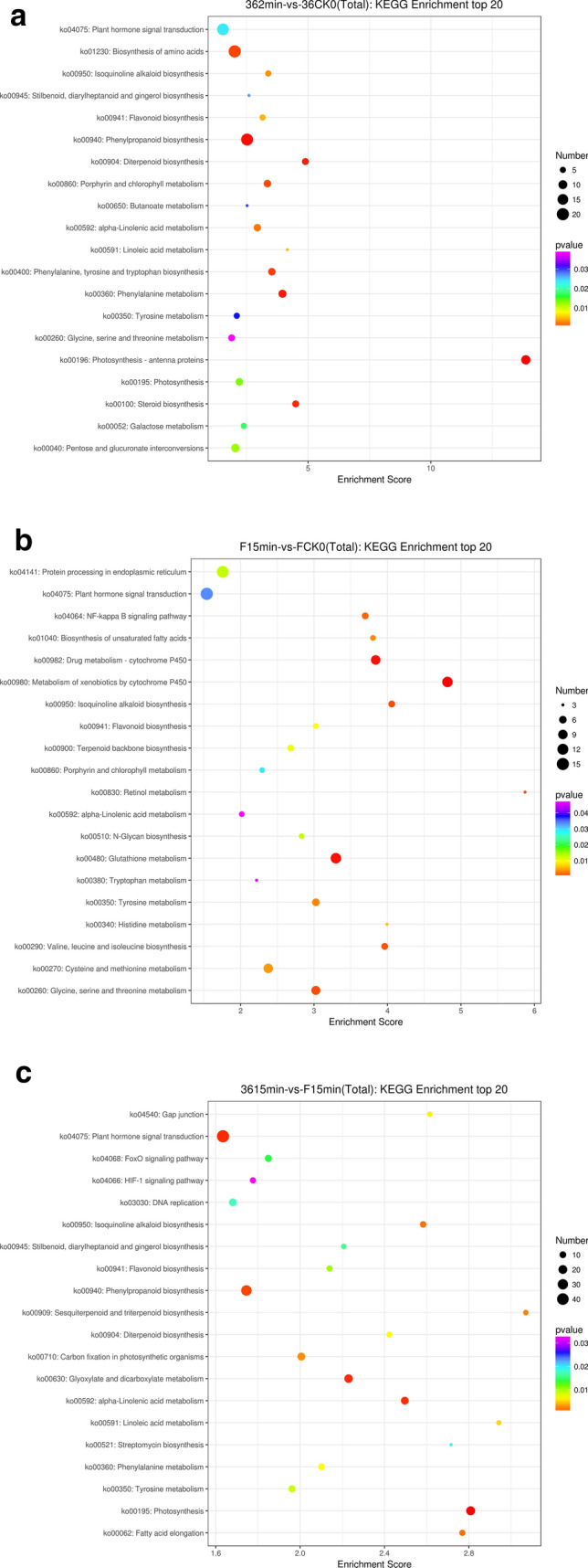
(**a**) KEGG enrichment of the ‘36’ 2 min vs ‘36’ CK0 groups. (**b**) KEGG enrichment of the ‘F’ 15 min vs ‘F’ CK0 groups. (**c**) KEGG enrichment of the ‘36’ 15 min vs ‘F’ 15 min groups. The figures was drawn with DEseq (v3.5.1). (http://www.bioconductor.org/packages/3.8/bioc/html/DESeq.html).

To further identify important metabolic pathways, we used KEGG pathway analysis to determine the biological functions of DEGs. Pathway entries with a number of corresponding DEGs greater than 2 were screened and sorted by the − log10 *p*-value of each entry. Phenylpropanoid biosynthesis (ko00940), phenylalanine metabolism (ko00360), tyrosine metabolism (ko00350), glutathione (GSH) metabolism (ko00480), flavonoid biosynthesis (ko00941), linoleic acid metabolism (ko00591), as well as starch and sucrose metabolism (ko00500) were found in the top 20 enrichment biological pathways of these compared. Phenylpropanoid biosynthesis (ko00940), phenylalanine metabolism (ko00360), tyrosine metabolism (ko00350), and GSH metabolism (ko00480) were the more significant pathways for enrichment among the three groups, which had a small *p*-value (*p* < 0.05) compared to that of the other pathways (Fig. 5a–c). The top 20 KEGG enrichment analysis of DEGs in all pairwise comparisons are shown in Supplementary Table S4.

### Identification of potential genes associated with enzymatic browning

**Figure 6:**
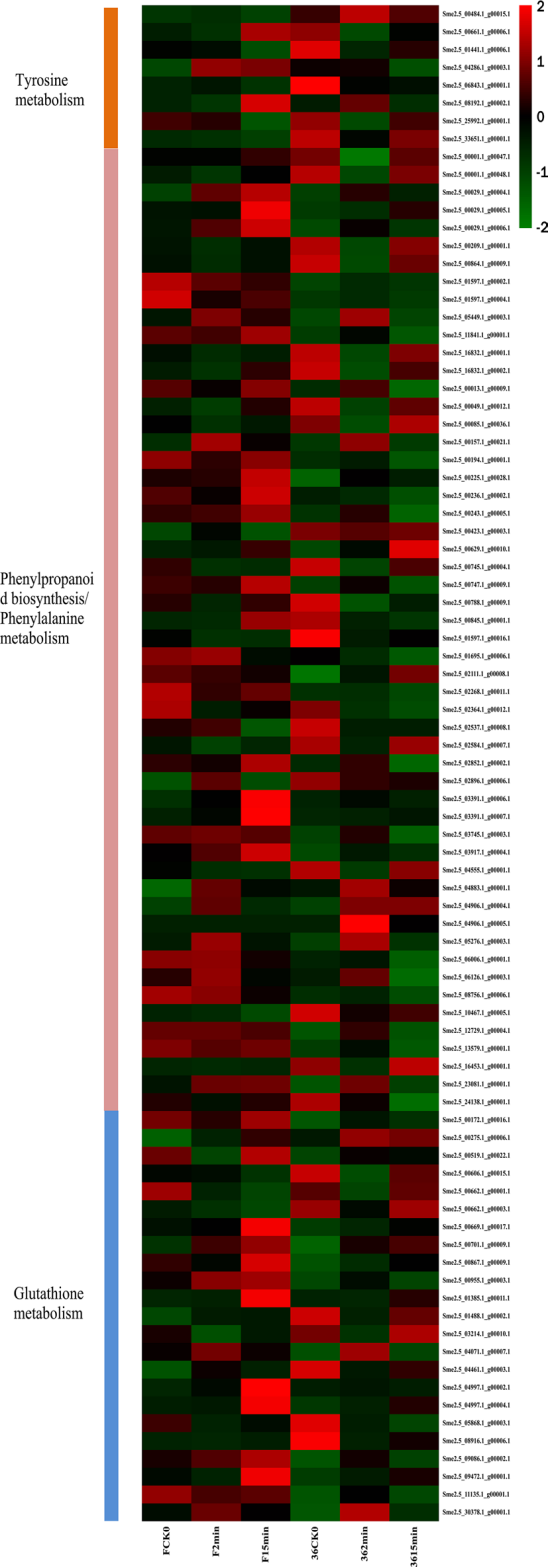
The heat maps for the browning-related genes. The red color in the picture indicates high expression genes, while the green color indicates low expression genes. The heat map was drawn with pheamap (v1.0.12) (Kolde, R. pheatmap: Pretty Heatmaps, r package version 1.0.12 (2019). https://CRAN.R-project.org/package=pheatmap).

Changes in the transcriptome of the eggplant after fresh-cut treatment were examined by cluster analysis of the gene expression patterns, which were presented in a heat map (Fig. 6). We analyzed the DEGs involved in the tyrosine metabolism pathway. There were 10 genes in total, including 7 PPO genes, 4 of which encoded PPO and 3 encoded catechol oxidase. Two of the four PPO encoding genes (*Sme2.5_25992.1_g00001.1*, *Sme2.5_11776.1_g00001.1*) were significantly up-regulated in ‘36’ vs ‘F’, while two genes (*Sme2.5_08192.1_g00002.1*, *Sme2.5_04286.1_g00003.1*) had the opposite result (Fig. 6). Furthermore, three genes encoding catechol oxidase (*Sme2.5_33651.1_g00001.1*, *Sme2.5_01441.1_g00006.1*, *Sme2.5_06843.1_g00001.1*) were significantly up-regulated in ‘36’ vs ‘F’. In the phenylpropanoid biosynthesis/phenylalanine metabolism pathway (ko00940/360), 64 DEGs were identified, including 22 POD-encoding genes and 3 PAL-encoding genes (*Sme2.5_00864.1_g00009.1*, *Sme2.5_16832.1_g00001.1*, *Sme2.5_16832.1_g00002.1*) (Fig. 6). This confirmed that the expression pattern of most of these genes were consistent. In total, 28 DEGs were found to be involved in GSH metabolism (ko00480), including 18 GST-encoding genes and 10 genes encoding L-ascorbate oxidase, most of which were down-regulated in ‘36’ vs ‘F’. In addition, 2 CAT-encoding DEGs were identified (Fig. 6). These results showed that the gene expression patterns of ‘36’ and ‘F’ were obviously changed after fresh-cut treatment.

### Identification of transcription factor genes

**Figure 7:**
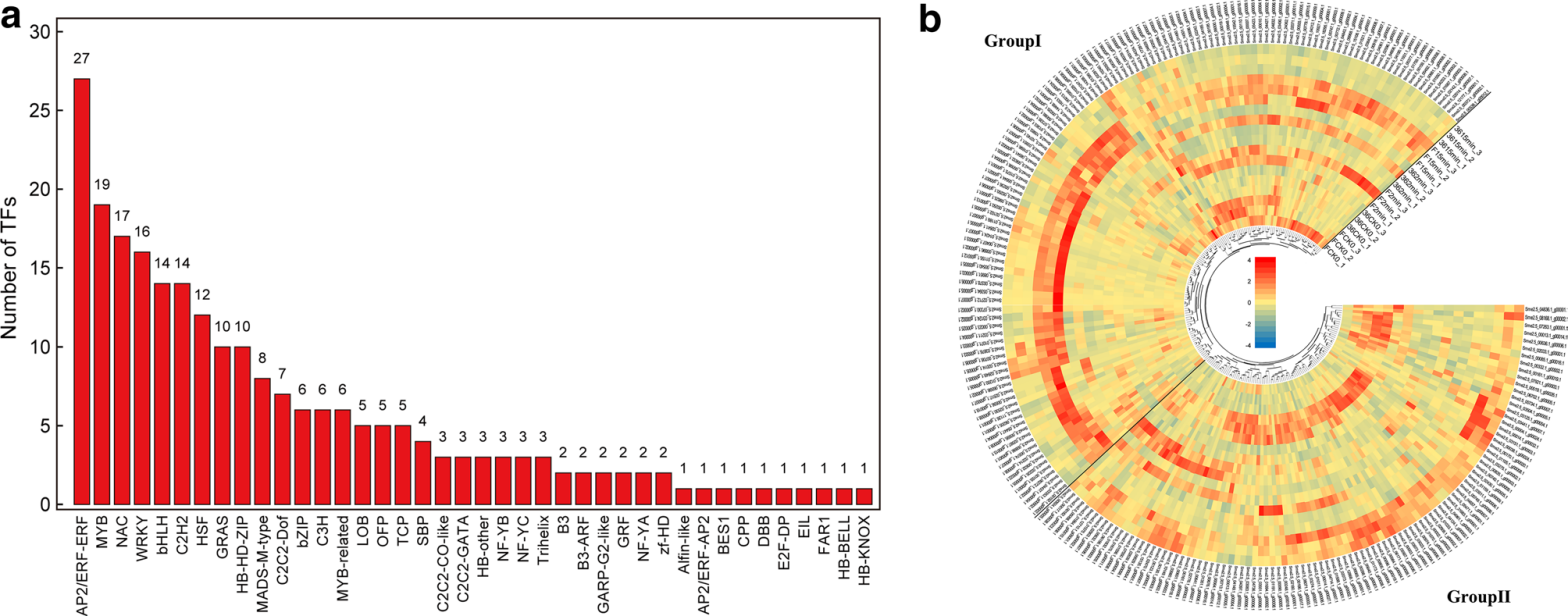
(**a**) Number of transcription factors (TFs) involved in fresh-cut eggplant browning. (**b**) Heat map presenting the expression patterns of different TFs in response to diverse treatments. The heat map was drawn with pheamap (v1.0.12) (Kolde, R. pheatmap: Pretty Heatmaps, r package version 1.0.12 (2019). https://CRAN.R-project.org/package=pheatmap).

To screen for TFs that may be involved in the enzymatic browning process in fresh-cut eggplant, we analyzed the structures of TFs that were differentially abundant in the ‘F’ and ‘36’ cultivar transcriptomes and detected 231 differential TFs from 40 TF families (Fig. 7a), including genes encoding 27 ERF TFs, 19 MYB TFs, 17 NAC TFs, 16 WRKY TFs, 14 bHLH TFs, 14 C2H2 TFs, 12 HSF TFs, and 10 GRAS TFs, among other types of TFs (Fig. 7a). Subsequently, the TF gene expression levels following the various treatments were analyzed, and the genes were grouped into the following two groups (Fig. 7b): Group I, in which the expression levels of most TFs were higher in ‘F’ than in ‘36’, and Group II, which exhibited the opposite expression pattern. These results imply that the complex browning process of fresh-cut eggplant may be regulated by these candidate TFs.

### Gene regulation network construction

**Figure 8:**
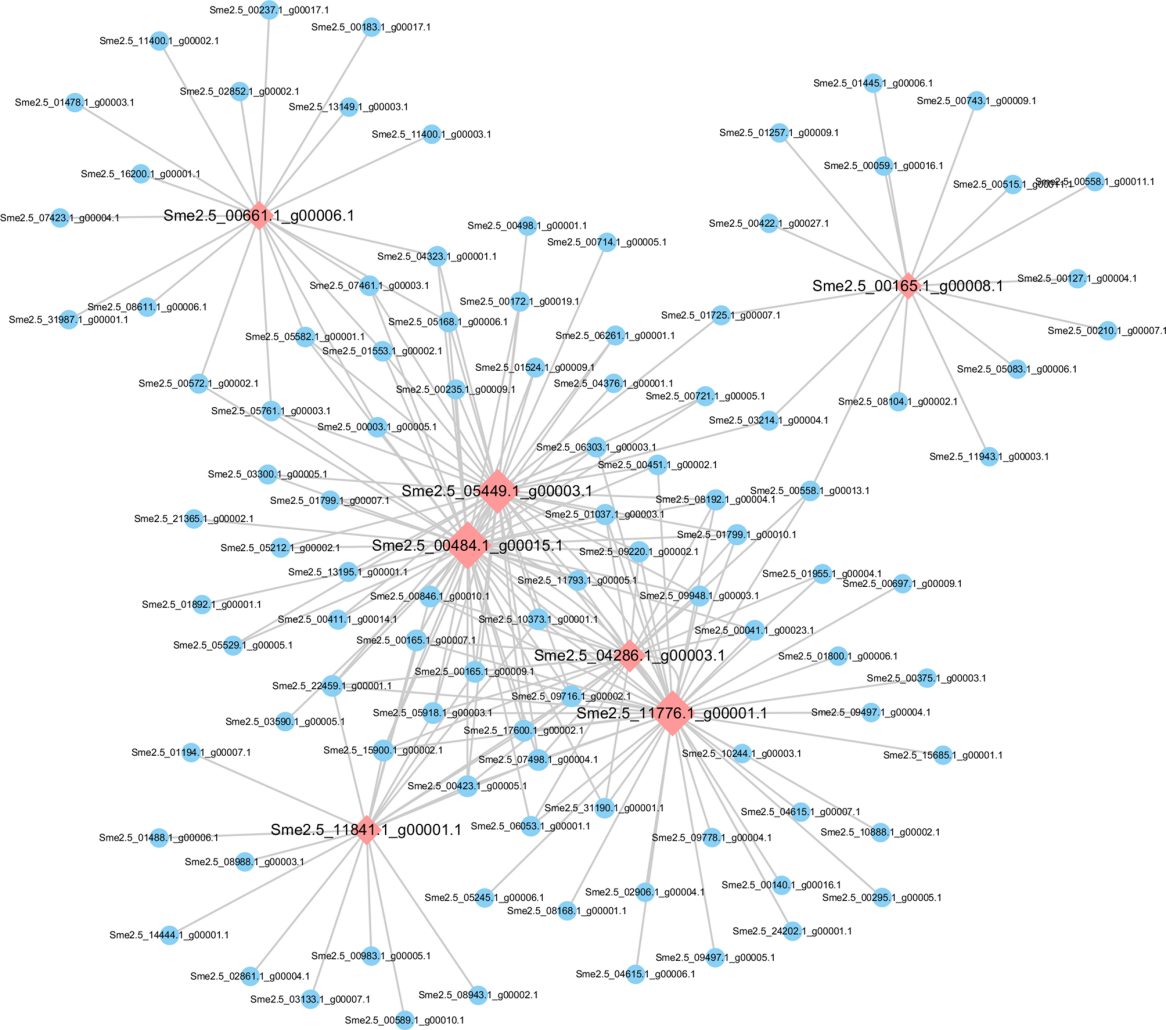
Gene regulatory networks for the KEGG pathway ko00350 (tyrosine metabolism). Cytoscape v3.6.0 (https://cytoscape.org/) was used to create the illustrations.

**Figure 9:**
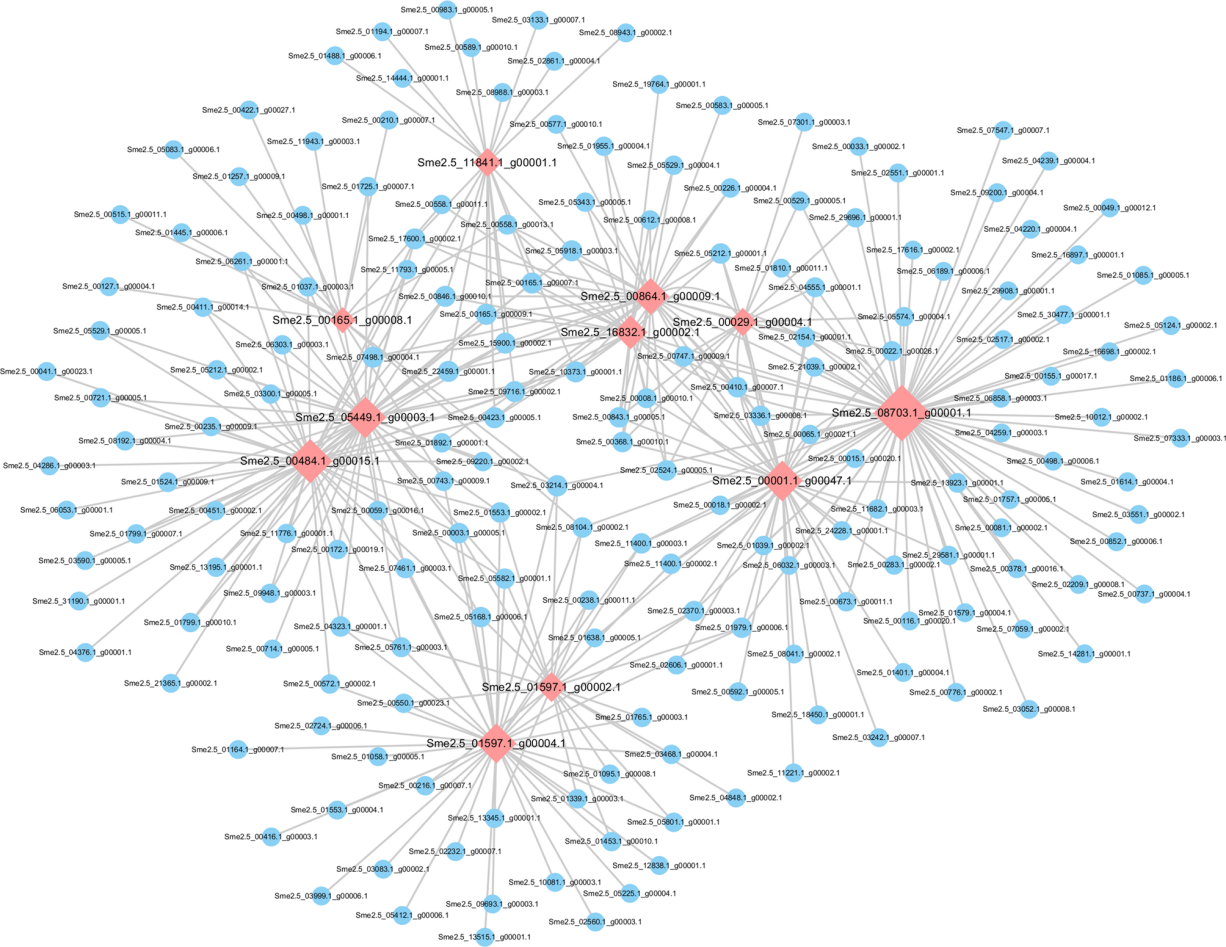
Gene regulatory networks of the KEGG pathway ko00360 (phenylalanine metabolism). Cytoscape v3.6.0 (https://cytoscape.org/) was used to create the illustrations.

The gene regulation network data for *Solanum lycopersicum*, *Solanum tuberosum*, *Arabidopsis*, and *Oryza sativa* L. were downloaded from the STRING database. The KEGG pathways ko00350 (tyrosine metabolism) and ko00360 (phenylalanine metabolism) were targeted, respectively. BLAST comparisons were used to construct the eggplant gene regulation network based on the similarities between eggplant and these four types of plants. In the gene regulatory network map constructed based on ko00350 (tyrosine metabolism), a total of 212 were interacting with each other, and 7 core genes (*Sme2.5_00661.1_g00006.1*, *Sme2.5_05449.1_g00003.1*, *Sme2.5_00484.1_g00015.1*, *Sme2.5_04286.1_g00003.1*, *Sme2.5_11776.1_g00001.1*, *Sme2.5_11841.1_g00001.1*, and *Sme2.5_00165.1_g00008.1*) were differentially expressed in the transcriptome data (Fig. 8). Among them, two genes were annotated as PPO (*Sme2.5_04286.1_g00003.1*, *Sme2.5_11776.1_g00001.1*); and *Sme2.5_00165.1_g00008.1* was annotated as tyrosine aminotransferase (Fig. 8). Furthermore, a gene regulatory network based on ko00360 (phenylalanine metabolism) was obtained for a total of 370 genes, including 11 core genes, among which 9 genes were differentially expressed in the transcriptome data (*Sme2.5_11841.1_g00001.1*, *Sme2.5_00864.1_g00009.1*, *Sme2.5_00029.1_g00004.1*, *Sme2.5_16832.1_g00002.1*, *Sme2.5_05449.1_g00003.1*, *Sme2.5_00484.1_g00015.1*, *Sme2.5_00001.1_g00047.1*, *Sme2.5_01597.1_g00002.1*, and *Sme2.5_01597.1_g00004.1*) (Fig. 9). Furthermore, *Sme2.5_16832.1_g00002.1* was annotated as PAL and *Sme2.5_00001.1_g00047.1* was annotated as trans-cinnamate 4-monooxygenase (Fig. 9).

### mRNA regulatory network associated with PPO genes

**Figure 10:**
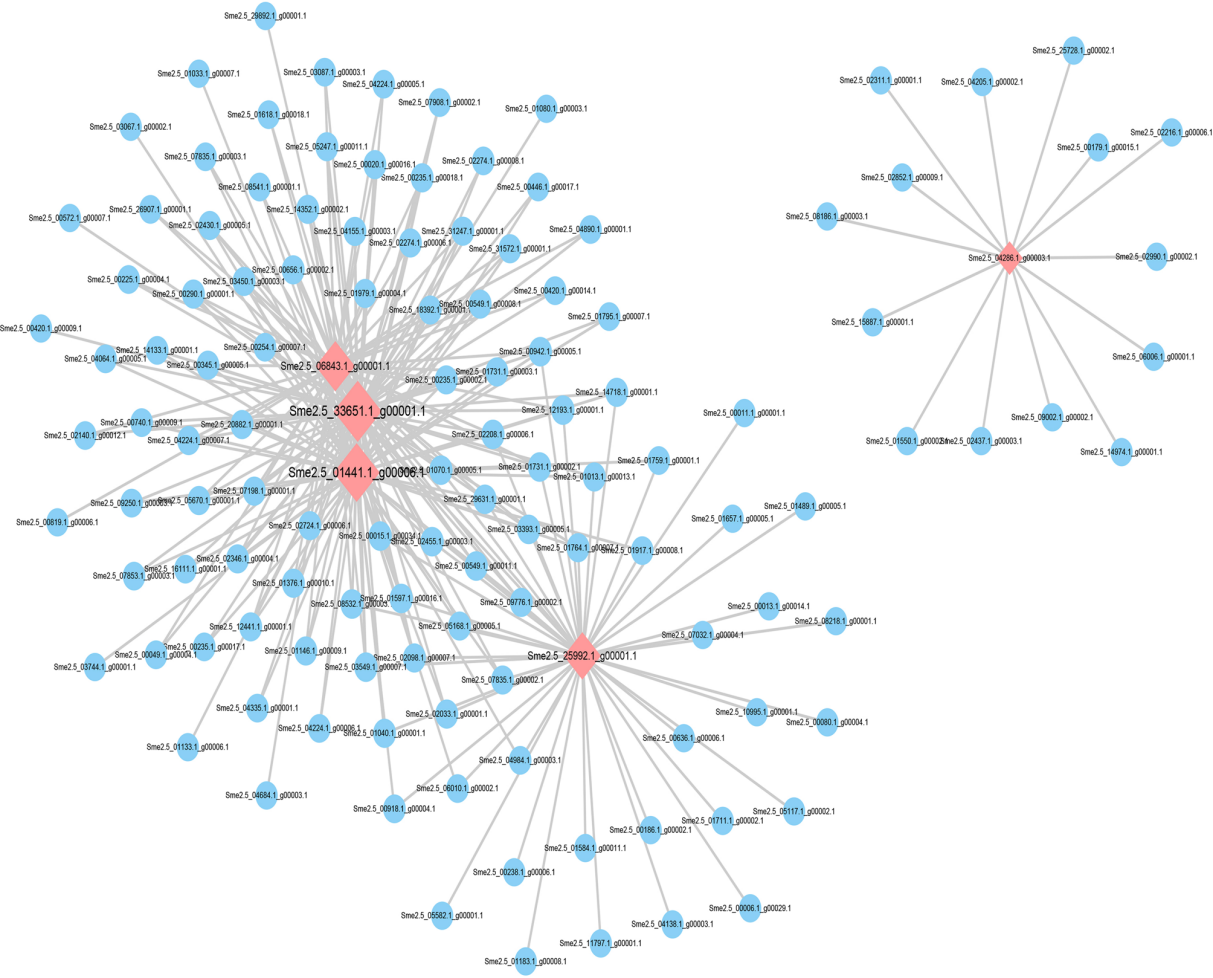
mRNA regulatory network associated with polyphenol oxidase (PPO) genes. Cytoscape v3.6.0 (https://cytoscape.org/) was used to create the illustrations.

Pearson correlation coefficient was calculated between all the mRNAs and four significant differentially expressed PPO genes (*Sme2.5_06843.1_g00001.1*, *Sme2.5_25992.1_g00001.1*, *Sme2.5_33651.1_g00001.1*, *Sme2.5_04286.1_g00003.1*). The mRNAs with correlation coefficient greater than 0.9 was considered to be genes that are co-expressed with the PPO genes, of which a total of 212 mRNA correlation coefficients met this criteria (Fig. 10). These related DEGs, such as zinc finger protein WIP4 (*Sme2.5_01070.1_g00005.1*) and wound-induced proteinase inhibitor 2 (*Sme2.5_04984.1_g00003.1*), may have a synergistic effect with PPO and participate in the browning process of fresh-cut eggplant, requiring further study.

### Validation of DEGs with quantitative real-time PCR (qRT-PCR)

**Figure 11:**
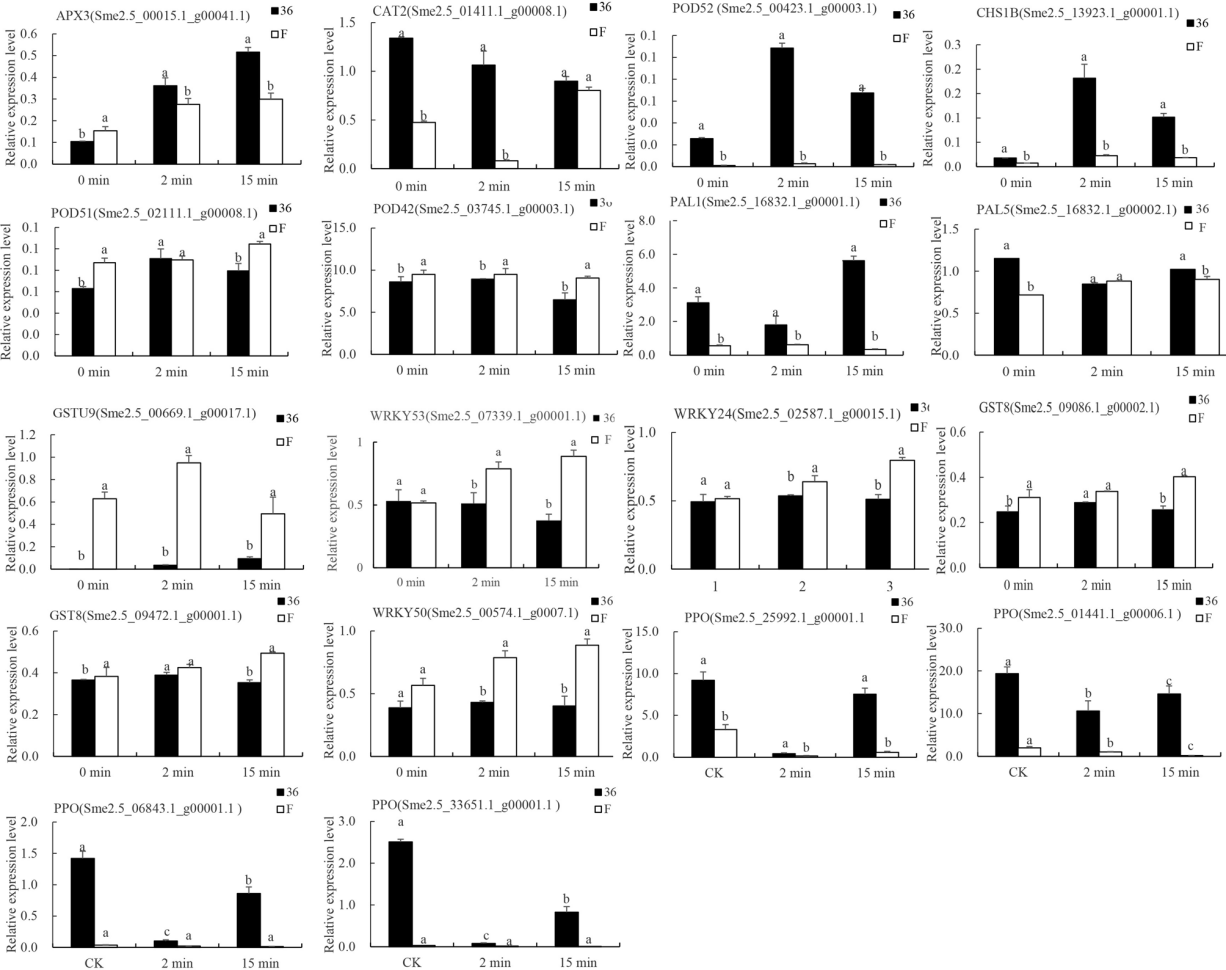
qRT-PCR of the expression levels of 18 browning-related genes. The error bars represent the standard error of three biological replicates. PKG was used as the internal control.

The heat map for the browning-related genes indicated that the expression levels varied dependent on both the cultivar and treatment (Fig. 6). To confirm the accuracy of the RNA-seq data, 18 browning-related genes were selected, and their expression levels investigated by qRT-PCR. The list of primer sequences are shown in Supplementary Table S5, whereas the quantitative results are shown in Fig. 11. The expression of DEGs were consistent with the transcriptome. As verified by qRT-PCR, the expression levels of PPO, POD, and PAL encoding genes in ‘36’ were significantly higher than that in ‘F’, whereas the activity of antioxidant enzymes (APX, GST) showed an opposite trend. The results of qRT-PCR were consistent with the analysis of the transcriptome, confirming the reliability of the RNA-seq data.

### qRT-PCR of eggplant materials with different browning tolerance

**Figure 12:**
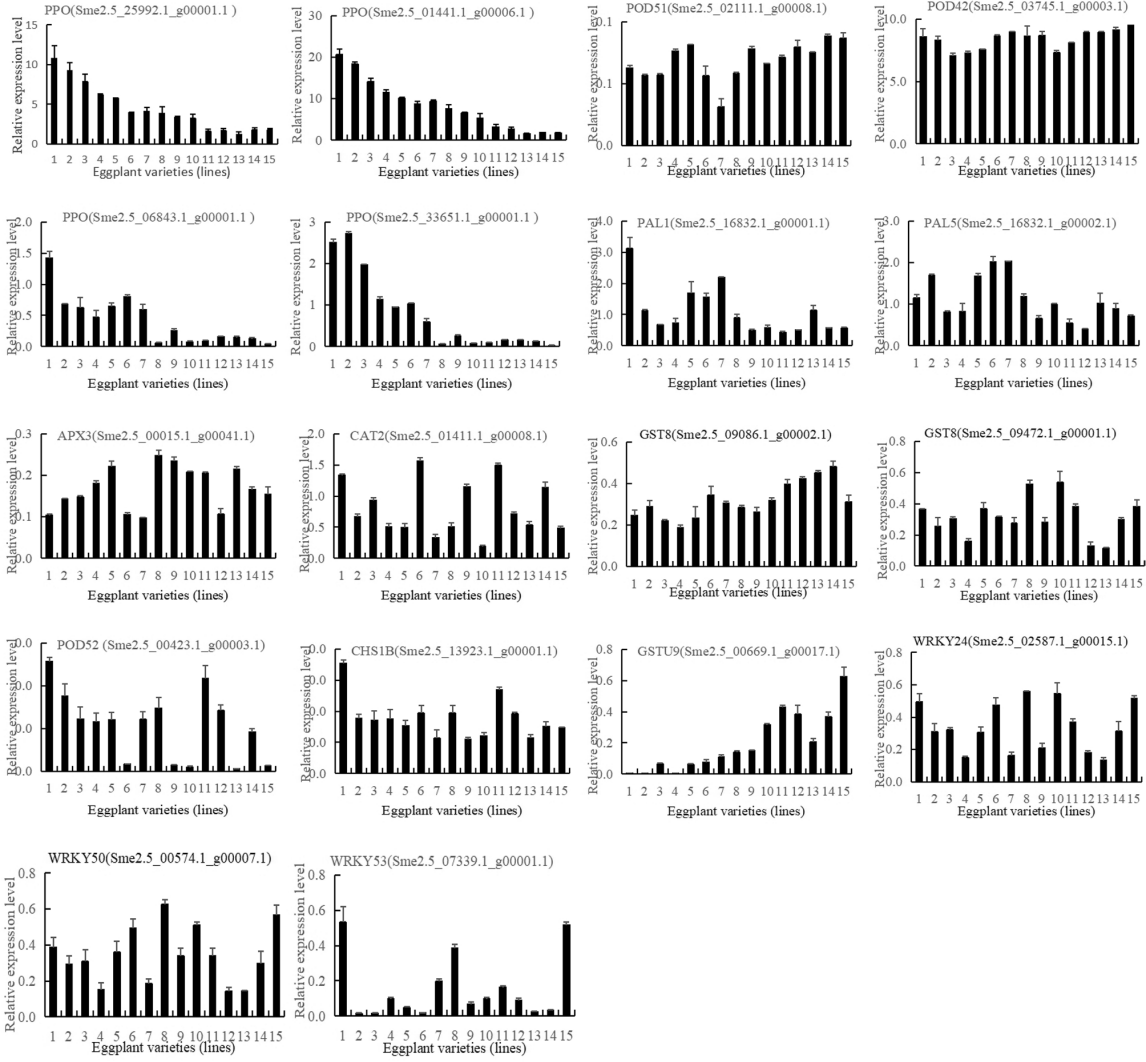
qRT-PCR of the expression levels of 18 browning-related genes in 15 eggplant materials with different browning tolerance.

We detected the expression levels of 18 browning-related genes in 15 eggplant materials with different browning tolerance by qRT-PCR. As shown in Fig. 12, the expression patterns of the four PPO-encoding genes were consistent, and the expression levels of all PPO-encoding genes were high in the browning-sensitive materials, while low in the browning-resistant materials. The expression levels of all PPO-encoding genes were the highest in the No. 1 browning-sensitive eggplant material (‘36’), and the lowest in the No. 15 eggplant material (‘F’) (Fig. 12). The genes related to the antioxidant enzymes, such as POD, CAT, APX, and GST, were found to be significantly differentially expressed during browning development. The expression levels of 3 POD DEGs, 1 CAT DEG, 1 APX DEGs, and 3 GST DEGs in browning-sensitive eggplant materials were lower than that in browning-resistant eggplant materials (Fig. 12). Furthermore, the expression pattern of the three WRKY TFs were consistent with the genes related to the antioxidant enzymes, suggesting their involvement in the antioxidant process (Fig. 12). Conversely, the DEGs associated with phenylpropanoid metabolism (2 PAL, 1 CHS) in browning-sensitive eggplant materials were higher than that in browning-resistant eggplant materials (Fig. 12).

### Activities of PPO, POD, PAL, SOD, and CAT

**Figure 13:**
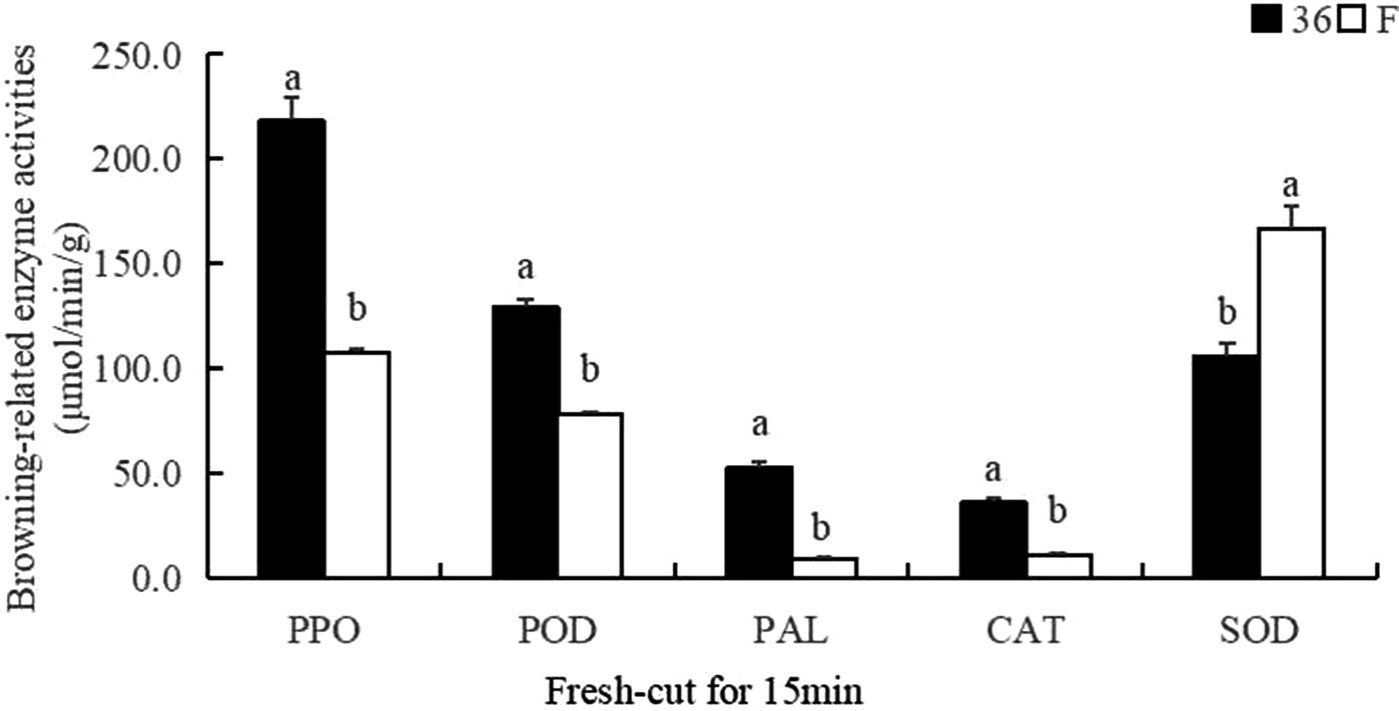
The enzyme activities of PAL, SOD, CAT, PPO, and POD in ‘36’ and ‘F’ after fresh-cut 15 min.

As shown in Fig. 13, the enzyme activities of PPO, POD, PAL, and CAT in ‘36’ were significantly higher than those of ‘F’ fresh-cut for 15 min. The increase of PPO activity is often accompanied by browning, and the degree of browning is positively correlated with the activity. Both PPO and POD play a major role in the browning of eggplant pulp, and they may play a synergistic role in the browning process. The increase of PAL activity promotes the accumulation of phenolic substrates and the occurrence of browning. SOD is a key enzyme in the cell membrane protection system, which can effectively remove the accumulation of intracellular reactive oxygen species (ROS) and the occurrence of browning. Therefore, the SOD activity of ‘F’ is significantly higher than that of ‘36’ (Fig. 13). The expression changes of *SmePPO*, *SmePOD*, *SmePAL*, and *SmeCAT* genes were consistent with the changing trend of the related enzymes (Fig. 6). The expression of related genes can be promoted during fruit browning, which can be translated into the corresponding enzymes to accelerate fruit browning.

## Discussion

Fresh-cut fruits and vegetables are ready-to-eat products made from fresh fruits and vegetables, which are cleaned, peeled, cut, dressed, packaged, and kept refrigerated. They are becoming more popular due to their convenience^21^. However, browning not only affects sensory quality, nutritional quality, and food quality of fruits and vegetables, which reduces commodity value, but also hinders product circulation, shortens the shelf life, and even seriously affects people's visual perception and appetite, making it unacceptable to consumers^15^. It has become one of the main obstacles restricting the development of fruits and vegetables storage, transportation, and processing. High-throughput sequencing analyses have revealed many genes related to the browning of vegetables and fruits^20^, but a more comprehensive understanding of gene expression during fresh-cut eggplant browning and strategies for browning control are still needed. The present study investigated the causes of browning and antioxidant capacity in a fresh-cut browning-sensitive cultivar (‘36’) compared to a browning-resistant cultivar (‘F’).

Enzymatic browning is considered the main reason for browning of fresh-cut fruits and vegetables. Enzymes related to phenolic oxidation (i.e., PPO, POD, CAT, and SOD) play a crucial role in the browning of fresh-cut fruits and vegetables. It is widely accepted that the enzymes encoded by the PPO and POD genes function together to induce browning^22^. Ali et al*.* found that the enzymatic browning of fresh-cut lotus root can be repressed by applying oxalic acid to decrease PPO and POD activity^23^. Similarly, an ascorbic acid (AsA) and *aloe vera* gel combined-treatment suppressed POD and PPO activities, providing indirect evidence that PPO and POD function together to influence the browning of fresh-cut lotus root^24^. Zheng et al*.* showed that the 0.1 mM melatonin treatment significantly reduced the expression of genes related to enzymatic browning and phenolic synthesis pathways, decreased POD and PPO activity, and thus reduced the surface browning of fresh-cut pear^25^. Furthermore, inhibition of PPO-encoding genes by artificial microRNAs in potato has been shown to lead to low PPO protein levels and low browning in potato tubers^26^. Gonzalez et al*.* found that specific editing of a single member of the *StPPO* gene family through the CRISPR/Cas9 system reduced PPO activity by 69% and enzymatic browning by 73% in potato tubers^27^, whereas evidence from another study indicated that overexpression of PPO in transgenic sugarcane led to higher PPO activity and darker juice color^28^. In the present study, 7 PPO-encoding genes involved in tyrosine metabolism and 22 POD-encoding genes were differentially regulated in ‘36’ and ‘F’. The expression level of most of the PPO-encoding genes in ‘36’ was significantly higher than that in ‘F’, and the expression level of PPO (*Sme2.5_25992.1_g00001.1*) was ~ 550-fold higher in ‘36’ than in ‘F’ (Fig. 6). It is generally accepted that the browning mechanism involves the interaction between the polyphenol substrate and PPO in the presence of oxygen, including two reaction processes: (1) hydroxylation of monophenols to diphenols, and (2) oxidation of diphenols to quinones. The *V*_*max*_ of the hydroxylation reaction is 1.8 mM/min and results in colorless products, while the *V*_*max*_ of the oxidation reaction is 24.5 mM/min with resultant coloration of quinones. The latter quinone reaction leads to the accumulation of melanin, which leads to browning of plant tissue. The specific structure of the polyphenol substrate determines the specific reaction time of plant tissue browning^29^. Additionally, the SOD and CAT enzymes play a pivotal role in the browning of fresh-cut fruits and vegetables. As the most important enzyme for scavenging free radicals, CAT can enhance the antioxidant properties of plants by catalyzing the degradation of H_2_O_2_^30^. UV-C treatment has been found to inhibit surface browning of fresh-cut apple by improving ROS metabolism and enhancing the activities of SOD and CAT^31^. Meanwhile, Zhou et al*.* reported that a methyl jasmonate treatment induced enzymatic browning of fresh-cut potato tubers and enhanced PPO, POD, and CAT activities^32^. In our study, two CAT-encoding genes were identified. It was significantly up-regulated in the browning-sensitive cultivar (‘36’) (Fig. 6). Therefore, we postulate that CAT enzyme activity increased rapidly when the plant tissue was cut and damaged, thus removing ROS and enhancing the oxidation tolerance.

Phenols are vital substrates of enzymatic browning in fresh-cut fruits and vegetables, with the type and content of phenolic compounds significantly determining browning characteristics^33^. Therefore, total phenolic content plays a major role in the browning of stored fruits. Phenolic levels have been found to increase in eight cultivars of eggplant during storage, whereas the phenolic concentration varied significantly among fresh-cut samples of eggplant cultivars^34^. In 2019, a study on the mechanism of the enzymatic browning reaction in potato processing showed that phenolic compounds are closely related to enzymatic browning of potato, and chlorogenic acid is an important substrate for enzymatic browning of potato^35^. The phenolic compounds identified in banana peels in recent years were mainly dopamine, followed by chlorogenic acid and coumarin, with dopaminerase oxidation being the main factor for banana browning^36^. Chlorogenic acid and epicatechin are the phenolic compounds with the highest contents in apple fruits^37^, while in caffeic acid and ferulic acid are the main substrates in mangos^38^. Mishra et al*.* also showed that browning of eggplant was related to the increased content of polyphenols^34^, and Liu et al*.* found that the variation of chlorogenic acid content in fresh-cut eggplant may be closely related to the browning of fresh-cut eggplant^39^. Biosynthesis of phenol compounds is involved in the pentose phosphate, shikimate, flavonoid, and phenylpropanoid pathways in plants^40^, which involve a variety of key enzymes, such as PAL, 4CL, CHS, F3′H, and COMT. PAL is an important enzyme in the phenylpropane metabolic pathway, catalyzing l-phenylalanine ammonia hydrolysis to generate trans-cinnamic acid, and then undergo a series of transformations to form various phenols, lignin, anthocyanins, alkaloids, and other compounds. The produced phenols provide a substrate for the browning reaction^41^. In a study of the expression of PAL and HSP in fresh-cut banana fruit, it was observed that mechanical injury (fresh-cutting) resulted in the accumulation of PAL mRNA in fruits, which led to a rapid increase in PAL activity. Mechanical injury also increased the synthesis rate of ethylene, which accelerated the decomposition of phenylalanine and the synthesis of a large number of phenolic substances. At this point, the phenolic compounds combined with PPO, activated by mechanical injury, to induce browning. Through linear regression analysis, it was found that the increase in PAL activity in fruit induced by the mechanical injury was highly correlated with the accumulation of phenolic substances^42^. Moreover, CHS is the key enzyme in anthocyanin biosynthesis^43^. Melatonin treatment at 0.1 mM increased the expression of PAL and CHS, enhanced their activities, and reduced the browning of fresh-cut pear fruit^25^. Studies of fresh-cut sweet potatoes treated with ultrasound have found that the induction of PAL was positively correlated with higher total phenolic content, thereby enhancing antioxidant capacity of the fresh-cut sweet potatoes against browning^44^. An 80% oxygen treatment was found to increase the activities of PAL and POD and the total phenolic content of fresh-cut potato, which effectively enhanced its antioxidant capacity^45^. Conversely, Gong et al*.* reported that browning inhibition has no observable correlation with either total phenol or POD activity^46^. Zhang et al*.* identified one PAL-encoding gene, one CHS-encoding gene, and two 4CL-encoding genes that were significantly up-regulated in Chinese walnut husks that browned, but another PAL-encoding gene was significantly up-regulated in anti-browning husks^47^. In the present study, many genes connected with phenylpropanoid biosynthesis/phenylalanine metabolism were differentially regulated in the browning-resistant cultivar compared with the browning-sensitive cultivar. Three PAL-encoding genes and one CHS-encoding gene were up-regulated in the browning-sensitive cultivar (Fig. 6). However, four COMT-encoding gene was down-regulated in the browning-sensitive cultivar (Fig. 6). These results suggest that fresh-cutting treatment induced the expression of the key enzyme genes in phenylalanine metabolism and flavonoid biosynthesis, and promoted the synthesis of polyphenols in plant cell tissues to improve their antioxidant capacity.

AsA and GSH also play important roles in maintaining the stability of proteins, structural integrity of the biofilm system, and defense against membrane lipid peroxidation. Together with antioxidant enzymes such as GST, APX, dehydro-AsA reductase (DAR), monodehydro-AsA reductase (MDAR), and GSH reductase (GR), the AsA-GSH circulatory system can effectively eliminate free radicals^48^. The coordinated operation of each component in the AsA-GSH cycle enables the accumulation of ROS in plants to be removed, which plays an important role in the enzymatic ROS removal mechanism of fresh-cut fruits and vegetables. Under normal conditions, the production and clearance of ROS in plants are in dynamic balance. When plants are subjected to mechanical stress, the content of ROS increases significantly. Excessive ROS will destroy biological molecules such as proteins and destroy the integrity and function of membranes. In the enzymatic defense system, GST catalyzes the reaction of GSH with membrane lipid peroxides, thus reducing the damage to the membrane structure caused by mechanical stress^49^. GST participates in the AsA-GSH cycle^50^, catalyzing H_2_O_2_ to generate oxidized GSH (GSSG) and H_2_O, and decompose organic hydroperoxides into alcohols, H_2_O, and GSSG, which are then reduced by GR to GSH^51^. With AsA as the electron donor, APX catalyzes the reduction of H_2_O_2_ to MDHA and H_2_O. MDHA has two reduction pathways: (1) reduced to AsA by MDHAR and (2) reduced to dehydro-AsA (DHA) and then reduced to AsA through DHAR with reduced GSH as an electron donor^52^. AsA is involved in the oxidation protection system against free radicals; it is easily soluble in water and has sensitive antioxidant properties. Therefore, a change in its content level is considered an important indicator for monitoring fruit and vegetable quality^53^. l-Ascorbate oxidase plays a catalytic role in physiological changes, which can oxidize AsA to DHA and reduce H_2_O_2_, thus eliminating free radicals in cells and delaying senescence^54^. Browning and PPO activities of fresh-cut artichoke bottoms were significantly inhibited by Cg supplemented with AsA^55^. Salminen et al*.* found that 3% AsA and 0.1% green tea extract prevented the browning of fresh-cut apple slices for up to 14 days^56^. GST is present in almost all plants with up to 90 genes encoding GST in plants. Most of these genes are differentially expressed under stress induction and play an important role in the mechanism of enzymatic ROS scavenging^57^. We found that the expression levels of 18 GST-encoding genes and 10 genes encoding l-ascorbate oxidase involved in GSH metabolism were significantly higher in the browning-resistant cultivar compared with the browning-sensitive cultivar for all treatments (Fig. 6), indicating that the browning-resistant cultivar had a stronger antioxidant capacity. This could explain why the fruit did not easily brown after cutting.

The browning of fruits and vegetables is closely related to ROS metabolism in plants^58^. WRKY TFs play an vital role in regulating various physiological processes in plants, including plant growth and development, as well as responses to biological, abiotic, and oxidative stress^59^. Furthermore, Wang et al*.* revealed that WRKY TF plays an important induction role in the ROS metabolic pathway^60^. Overexpression of ATWRKY30 increased the activity of antioxidant enzymes (i.e., CAT, SOD, POX, and APX) in wheat plants^61^. Zhang et al*.* ported that one WRKY TF was differentially regulated in the fresh-cut browning and white husks of Chinese walnut^47^. There were four WRKY TFs significantly differentially expressed in fresh-cut luffa fruit^20^. These findings provide direct evidence that WRKY TFs play a crucial role in fresh-cut vegetables and fruits. In the current study, 16 WRKY TFs was observed and the expression level of most of the WRKY TFs were significantly up-regulated in the browning-resistant cultivar compared with the browning-sensitive cultivar (Fig. 6). The relationship between WRKY TFs and fresh-cut eggplant browning was revealed in our study. In addition, there are other TFs, such as MYB, ERF, NAC, and bHLH, that show different levels of transcription. The changes of these TFs may be involved in the browning process of fresh-cut eggplant. However, further studies on their structural and functional properties are needed to determine their potential influence on the browning of fresh-cut eggplant fruits.

Gene expression is often affected by other genes, thus forming a complex regulatory network of interaction and influence between genes. Gene regulatory network construction is an important technique in bioinformatics and systems biology that can be used to comprehensively study the mechanisms underlying cellular and metabolic processes by integrating gene expression data, biological information, computational analysis, and mathematical models. Furthermore, analysis of gene regulatory networks can allow researchers to explore the origin and evolution of life^62^. We set up two gene expression regulatory networks based on KEGG pathways ko00350 (tyrosine metabolism) and ko00360 (phenylalanine metabolism), respectively. We screened some core genes that showed differentially expressed transcriptome results, such as *Sme2.5_16832.1_g00002.1*, which was annotated as PAL and the *Sme2.5_00001.1_g00047.1*, which was annotated as trans-cinnamate 4-monooxygenase (Figs. 8 and 9). They play an important regulatory role in phenylalanine metabolism involved in phenolic synthesis. Moreover, a total of 212 mRNA were co-expressed with 4 PPO genes (correlation coefficient greater than 0.9) by Pearson correlation coefficient (Fig. 11), suggesting that they may be involved in the process of fresh-cut eggplant browning. However, further studies on their structural and functional properties are needed to determine their potential influence on the browning of fresh-cut eggplant fruits.

Finally, we detected the expression levels of 18 browning-related genes in 15 eggplant materials with different browning tolerance by qRT-PCR (Fig. 12). The results showed that the transcriptional expression of browning related genes was variety specific. These results suggest that these genes regulate the browning process of fresh-cut eggplant by participating in these biological processes, but further study is still needed for confirmation.

## Conclusion

62 genes from six gene families (i.e., PPO, PAL, POD, CAT, APX, and GST) were differentially regulated in fresh-cut eggplant fruit from transcriptoms and the enzyme activities of PPO, POD, PAL, and CAT in ‘36’ were significantly higher than ‘F’ for 15 min, while SOD was down-regulated. Two gene expression regulatory networks was set up based on tyrosine metabolism (ko00350) and phenylpropanoid biosynthesis (ko00940/360) and 16 genes were differentially expressed in transcriptomic data. 18 browning related genes randomly screened in 15 eggplant materials with different browning tolerance showed variety specific.

## Material and methods

### Plant materials

The 15 eggplant materials were obtained from a breeding line produced by our lab at the Shanghai Key Laboratory of Protected Horticultural Technology of the Shanghai Academy of Agricultural Sciences, Shanghai, China. Mature commercial fruits with the same growth trend, uniform size, no diseases and insect pests, and no obvious mechanical damage were selected for the experiment. All plant studies involving (*Solanum melongena* L.) were carried out in accordance with relevant institutional, national, and international guidelines and legislation.

### Preliminary screening of eggplant materials with different browning tolerance

Fresh-cutting treatment was performed on more than 60 eggplant materials planted in the Zhuanghang Experimental Station of Shanghai Academy of Agricultural Sciences, and the browning occurrence time of each material was recorded. Specifically, 15 eggplant materials were preliminary screened (as shown in Table 2), and subsequent experiments were conducted. In the subsequent experiments, all the eggplant material names were replaced by the numbers in Table 2. Finally, the eggplant cultivars ‘36’ was considered as the most browning-sensitive, while ‘F’ was considered as the most browning-resistant.

**Table 2:** The 15 eggplant variants (lines) selected from preliminary screening.

No	Variant (line)	Browning starting time
1	36	< 2 min
2	E3-2-2-1-2-2-1	
3	E-3-6-1-1-1-1	
4	308-1-4-3-2-1-2-1	
5	Tian jiao 2-2-1-1-1	
6	Hong niang-1-1-2	2 min < T < 15 min
7	Hang zhou hong qie-2-2-1-1	
8	E2-2-2-1-2-2-1	
9	E2-1-2-3-3-1	
10	Hu qie五	
11	E-2-6-2-2-3-1-1	> 15 min
12	308-1-3-1-1-4-1-1	
13	Jiang qie-1	
14	308-2-2-1-2-1-1-1	
15	F	

### Fresh-cut treatments

The eggplant cultivars ‘36’ (browning-sensitive) and ‘F’ (browning-resistant) were obtained from a breeding line produced by our lab at the Shanghai Key Laboratory of Protected Horticultural Technology of the Shanghai Academy of Agricultural Sciences, Shanghai, China. The fruit samples were carefully peeled with a stainless-steel knife. Three treatments were established based on the time elapsed since cutting: control, fresh-cut for 0 min (36 CK0 and F CK0, respectively); treatment 1, fresh-cut for 2 min (36 2 min and F 2 min, respectively); and treatment 2, fresh-cut for 15 min (36 15 min and F 15 min, respectively). After the samples were left for the prescribed amount of time, they were frozen in liquid nitrogen and kept at − 80 °C until RNA extraction. Moreover, after peeling, 15 eggplant materials were immediately sampled and frozen in liquid nitrogen for qPT-PCR.

### Total RNA extraction, library construction, and RNA sequencing

Total RNA was isolated using a mirVana RNA Isolation Kit (Ambion-1561; Thermo Fisher Scientific, Waltham, MA, USA) according to the manufacturer’s protocol. The concentrations and quality of the RNA samples were assessed using a NanoDrop ND-2000 spectrophotometer (Thermo Fisher Scientific). The A260/280 ratios of individual samples were all above 2. The 28S/18S ratio and RIN values were determined using an Agilent 2100 system (Agilent, Santa Clara, CA, USA). A TruSeq Stranded mRNA LT Sample Prep Kit (Illumina, San Diego, CA, USA) was used to construct the sequencing library, and then samples were sequenced using the HiSeq 2500 system (Illumina) after quality control analysis using an Agilent 2100 Bioanalyzer.

### RNA sequence analysis and DEG identification

Raw data (raw reads) were processed using Trimmomatic v0.36^63^. The reads containing ploy-N and low-quality reads were removed to obtain clean reads. Then, the clean reads were mapped to the reference genome using HISAT2^64^. The reads were reassembled using StringTie v2.1.7^65^. Then, gene structure extension and novel transcript identification were performed by comparing the reference genome and the known annotated genes using Cuffcompare v8^66^.

The gene expression level was indicated by a fragments per kb per million (FPKM) value^67^. To calculate the different expression levels of genes among the samples, the htseq-count^68^ was used to acquire the number of reads in each sample. Two functions (estimatSizeFactors and nbinomTest) in the DESeq^69^ R package (http://www.bioconductor.org/packages/3.8/bioc/html/DESeq.html) were used to normalize the data and calculate the *p*-value and fold-change. A *p*-value < 0.05 and fold-change > 2 or < 0.5 were set as thresholds for significantly differential expression.

### Functional annotation and classification of DEGs

The sequences of the DEGs were aligned to the KEGG database using BLASTX, retrieving proteins with the highest sequence similarity with the given sequences along with functional annotations for their proteins^70^. GO annotations of the DEGs were obtained using Blast2GO v6.0^71^. Web Gene Ontology Annotation Plot v2.0 was used to perform GO functional classification with a Pearson’s chi-square test^72^. The DEGs were mapped to GO terms according to the analyses, and the number of DEGs in each term was calculated^73^.

### Quantitative real-time PCR validation

qRT-PCR was performed to verify the accuracy of the gene expression profile obtained from the RNA-seq data. Total RNA was extracted using the Quick RNA isolation Kit (Beijing Tsingke Biotech, Beijing, China) according to the manufacturer’s protocol. First-strand cDNA was synthesized from 2 μg total RNA using M-MLV reverse transcriptase (Beijing Tsingke Biotech) and Oligo (dT)18 in a 25 μL reaction. Real-time PCR was performed with SYBR Green PCR mix (TaKaRa, Shiga, Japan). *SmPKG* was used as an endogenous control gene for qRT-PCR analyses. Relative expression levels of the target genes were calculated using the 2^−ΔΔCt^ method. The primers used are listed in Supplementary Table S5.

### Gene regulation network construction

The gene regulation network data for *Solanum lycopersicum*, *Solanum tuberosum*, *Arabidopsis*, and *Oryza sativa* L. were downloaded from the STRING database. BLAST comparisons were used to construct the eggplant gene regulation network based on the similarities between eggplant and these four types of plants. Cytoscape v3.6.0 (https://cytoscape.org/) was used to create the illustrations.

### Ethical standards

The conducted experiments comply with the laws of China

## Supplementary Information

Supplementary Figure S1.

Supplementary Table S1.

Supplementary Table S2.

Supplementary Table S3.

Supplementary Table S4.

Supplementary Table S5.

## Data Availability

Data supporting the results and conclusions are included in both the article and additional files. All the transcriptome data have been deposited in the NCBI Sequence Read Archive (SRA) under accession number: PRJNA679701 (http://www.ncbi.nlm.nih.gov/sra).
