# Supplementary material for: Transcriptome profiling reveals potential genes involved in browning of fresh-cut eggplant (Solanum melongena L.)
Source: Sci Rep. 2021 Aug 9;11:16081. doi: 10.1038/s41598-021-94831-z (PMC8352891; doi:10.1038/s41598-021-94831-z)
Supplement: Supplementary file 1 — Supplementary Figure S1. [file 41598_2021_94831_MOESM1_ESM.docx]

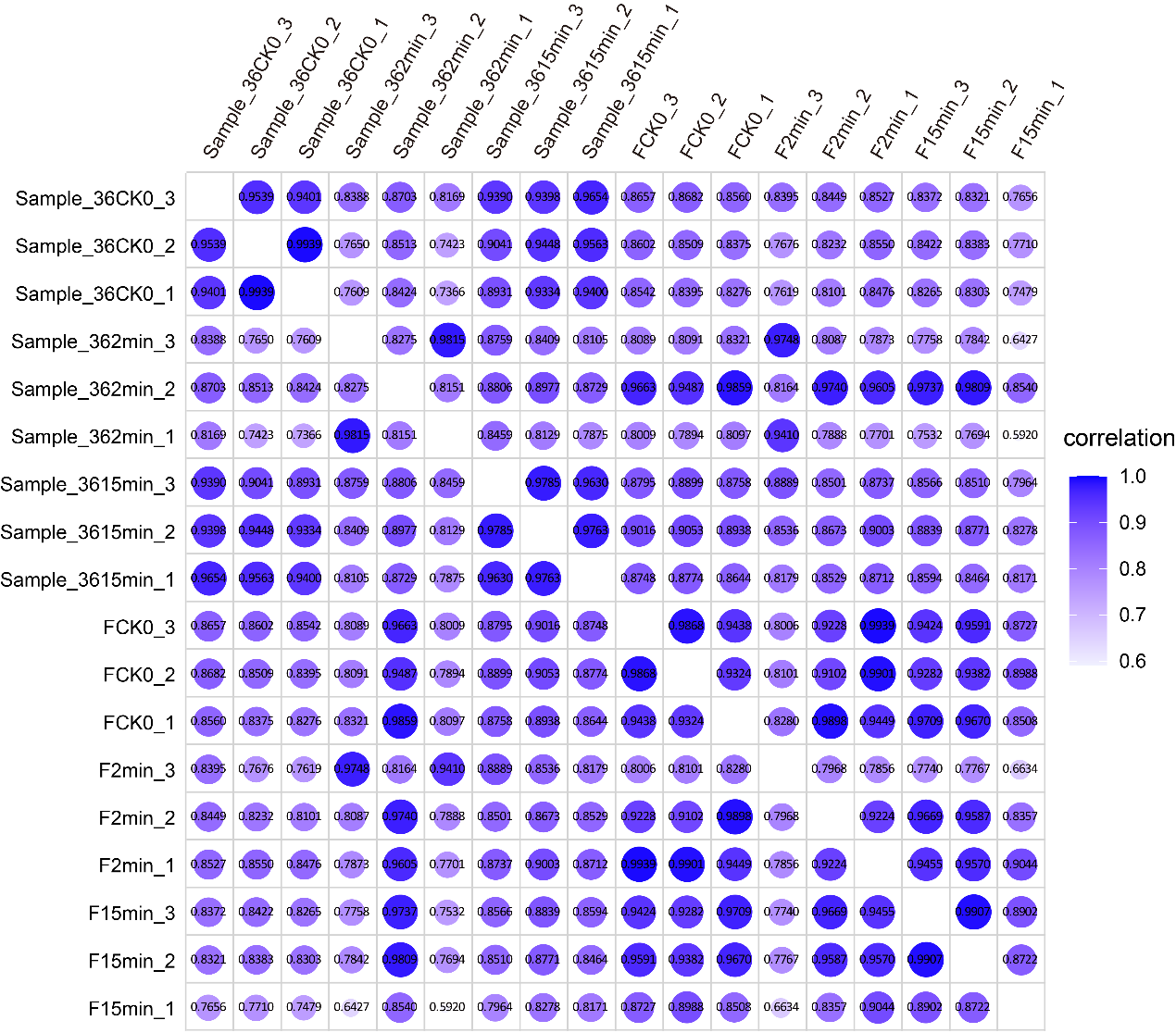


Supplementary Figure S1. Heatmap of Pearson's correlation coefficients between all pairs of samples. The heat map was drawn with DEseq (v3.5.1). http://www.bioconductor.org/packages/3.8/bioc/html/DESeq.html
